# Bacterial Colonization of the Condyle in Patients with Advanced Mandibular Osteoradionecrosis: Analysis of Hemimandibulectomy Specimens

**DOI:** 10.1155/2021/9998397

**Published:** 2021-11-22

**Authors:** Daisuke Takeda, Kazunobu Hashikawa, Manabu Shigeoka, Maki Kanzawa, Nanae Yatagai, Satomi Arimoto, Junya Kusumoto, Takumi Hasegawa, Hiroto Terashi, Masaya Akashi

**Affiliations:** ^1^Department of Oral and Maxillofacial Surgery, Kobe University Graduate School of Medicine, Kobe, Japan; ^2^Department of Plastic and Reconstructive Surgery, Nagoya University Graduate School of Medicine, Nagoya, Japan; ^3^Division of Pathology, Department of Pathology, Kobe University Graduate School of Medicine, Kobe, Japan; ^4^Division of Diagnostic Pathology, Department of Pathology, Kobe University Graduate School of Medicine, Kobe, Japan; ^5^Department of Plastic Surgery, Kobe University Graduate School of Medicine, Kobe, Japan

## Abstract

Advanced mandibular osteoradionecrosis (ORN) sometimes requires extended resection (e.g., hemimandibulectomy). Bacterial infection contributes to ORN pathogenesis. To control infection and determine the extent of debridement required, an understanding of bacterial spread within sites of mandibular ORN is important. The current study used a histopathological approach to assess bacterial colonization in the mandibular condyle and elucidate possible paths of bacterial spread towards the mandibular condyle. Four hemimandibulectomy specimens were selected. Areas of bone destruction were macroscopically assessed and confirmed using hematoxylin and eosin staining. Bacterial presence within mandibular condyle was confirmed with Gram staining. Bone exposure was observed in the molar area in all specimens. Macroscopic bone destruction was apparent especially near the medial side of the cortical wall. Gram staining revealed bacterial colonization of the mandibular condyle in three of the four specimens. In conclusion, bacteria tended to spread posteriorly and through the medial side of the mandibular cortical wall. In patients with advanced ORN, the potential for bacterial colonization of the mandibular condyle should be considered during treatment.

## 1. Introduction

Radiation therapy (RT) is an indispensable treatment option for the management of head and neck malignancies. Osteoradionecrosis (ORN) is one of the most serious oral complications of RT for patients with head and neck cancer [1]. ORN is defined as an area of exposed bone secondary to necrosis following RT, which fails to heal after 3–6 months [2]. ORN is considered secondary to bone and vascular damage, which causes a hypoxic-hypovascular-hypocellular environment (i.e., the “three H” principle) [3] and a late effect of a radiation-induced fibroatrophic process [4]. ORN is primarily accompanied by local infection; thus, the main goal of ORN treatment is the control of local infection [5].

ORN frequently occurs in the molar area of the mandible [6, 7]; it generally extends posteriorly (i.e., from the mandibular body to the ramus and condyle) but not anteriorly (i.e., to the mandibular symphysis). To the best of our knowledge, there have been few studies concerning bacterial spread within the mandible in patients with ORN, although this understanding is important for controlling infection and establishing an adequate extent of debridement for patients with advanced mandibular ORN.

Here, we assessed bacterial colonization in mandibular condyle specimens from patients who underwent hemimandibulectomy for treatment of advanced mandibular ORN. We demonstrate the presence of bacteria in the condyle of patients with severe mandibular ORN. Our findings will be useful in determining possible paths of bacterial spread from mandibular molar regions towards the condyle, based on coronal serial sections of hemimandibulectomy specimens.

## 2. Methods

### 2.1. Patients

Four consecutive patients with advanced mandibular ORN were included in this study. Their clinical characteristics were retrospectively obtained from hospital records: sex, age, pathological diagnosis, primary site, RT type, radiation dose, duration between RT completion date and operation, chemotherapy, and location of bone exposure, pathological fracture, cutaneous skin fistula, and preoperative inferior nerve paralysis. All patients had clinically exposed necrotic bone that failed to heal over a period of 3–6 months in the previously irradiated area, as well as repeated local infections. All patients had undergone hemimandibulectomy and simultaneous vascularized free fibula flap reconstruction at the Department of Oral and Maxillofacial Surgery and Plastic Surgery, Kobe University Hospital, during the period from January 2019 to December 2019. The extent of hemimandibulectomy with adequate safety margins (>10 mm) from apparent osteolytic areas was determined by preoperative thin-slice computed tomography (CT) imaging.

### 2.2. Pathological Specimen Preparation and Examination

Following hemimandibulectomy, all excised bone specimens were fixed in formalin and decalcified, although they were not frozen. Decalcification was performed as follows: formic acid (98%) (Wako, Osaka, Japan) was diluted to 10% in distilled water. Bone specimens were immersed in 10% formic acid with ion-exchange resin and treated by ultrasonic histoprocessor Histra-DC (Jokoh, Tokyo, Japan) for several weeks [8]. The bone specimens were cut in approximately 1.5 cm blocks and embedded in paraffin. Thin sections were obtained from these paraffin blocks and stained with hematoxylin and eosin for light microscopy analysis. The thin sections were also subjected to Gram staining using the Brown–Hopps method. All pathological sections were independently examined by two experienced pathologists (MS and MK). The extents of macroscopic bone destruction and bacterial colonization were each classified into four grades: −, none; ±, little; +, moderate; and ++, severe. The Ethics Committee of Kobe University Hospital approved this study (permission number: 180093), and all patients provided informed consent.

## 3. Results

The clinical characteristics of patients with mandibular ORN in this report are shown in Table 1. The primary disease was nasopharyngeal cancer in two patients (patients 1 and 4) and oropharyngeal cancer in two patients (patients 2 and 3). Three patients (patients 1, 2, and 4) received concurrent chemoradiotherapy in our hospital. One patient (patient 3) received conventional RT alone, following local tumor resection. Three patients (patients 1, 3, and 4) received conventional RT, while one patient (patient 2) received intensity-modulated radiotherapy. No patients underwent neck dissection.

Intraoral exposed bone was evident in molar regions in all patients. Two patients (patients 1 and 2) experienced severe neuropathic pain that interfered with daily life and caused sleep deprivation through the extension of necrosis to the inferior alveolar nerve; this led to pathologic fracture before surgery. One patient (patient 3) exhibited excessive bleeding from the inferior alveolar arteries and veins; therefore, transcatheter arterial embolization was performed urgently. The median interval between the end of RT and surgical treatment of ORN was 139 months (range, 43–201 months).

**Figure 1:**
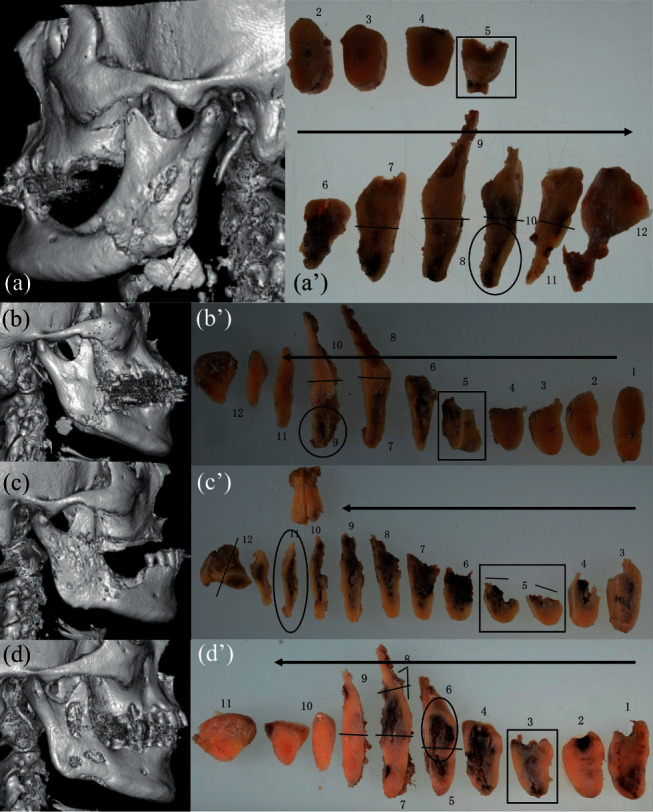
Lateral three-dimensional volume rendering computed tomography images (a–d) and macroscopic images of hemimandibulectomy specimens from patients with ORN (a'–d'). Black arrowheads indicate posterior directions. Black box indicates area of intraoral exposed bone. (a) Patient 1. (a') Black circle indicates macroscopic bone destruction of the medial side of cortical bone near the inferior margin of the mandible. (b) Patient 2. (b') Black circle indicates bone destruction of the medial side of cortical bone near the mandibular angle. (c) Patient 3. (c') Black circle indicates bone destruction of the medial side of cortical bone of mandibular ramus. (d) Patient 4. (d') Black circle indicates apparent bone destruction of the medial and lateral side of cortical bone of mandibular ramus.

Although preoperative CT in three patients (Figure 1(a), (c), and (d)) showed osteolysis of mandibular ramus cortices extending nearly to the condyle, osteolysis was detected only near the mandibular angle (Figure 1(b)). The extents of macroscopic bone destruction and bacterial colonization in the mandibular condyle are shown in Table 2. Coronal serial sections of hemimandibulectomy specimens revealed that macroscopic bone destruction with blackened degeneration was apparent in the medial sides of the cortical wall in patients 1, 2, and 4 (Figure 1(a'), (b'), and (d')). In patient 3, macroscopic bone destruction with blackened degeneration was present in whole areas of bone marrow in the mandibular body and ramus (Figure 1(c')).

**Figure 2:**
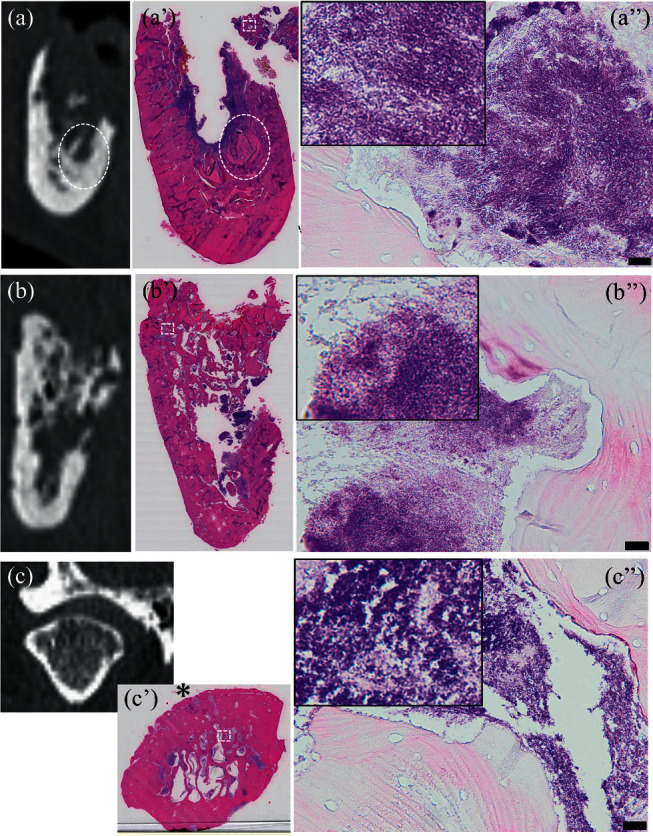
Preoperative CT images (a–c) and results of hematoxylin and eosin staining (a'–c') in patient 3. White dotted circle in (a) and (a') indicate mandibular canal. Results of Gram staining of areas indicated with white dotted boxes are shown in enlarged images (a”–c”). Severe bone destruction and inflammation in mandibular body (a') and ramus (b'). Apparent bacterial colonization is evident (a” and b”). Bone marrow hollowing and inflammatory cell infiltration (c'), as well as bacterial colonization (c”), are evident in mandibular condyle. Asterisk (*∗*) indicates the articular surfaces of condyle. Scale bar = 20 *μ*m.

**Figure 3:**
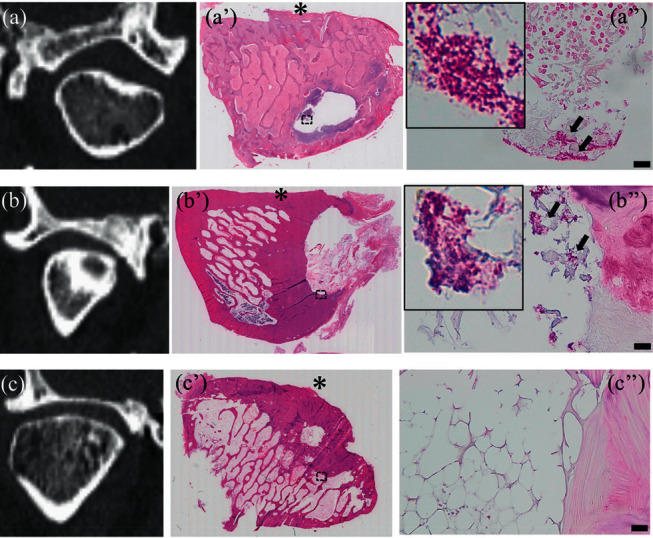
Preoperative CT images of condyles (a–c) and results of hematoxylin and eosin staining (a'–c') in mandibular condyles of patients 1, 2, and 4. Results of Gram staining of areas indicated with black dotted boxes are shown in enlarged images (a”–c”). Within the region of partial bone marrow hollowing, inflammatory cell infiltration (a') and bacterial colonization (a”) are evident in condyle of patient 1. Within the region of cortical hollowing, chronic fibrosis (b) and bacterial colonization (b”) are evident near condyle of patient 2. Although fibrosis and fatty degeneration are evident in bone marrow area (c'), no bacterial colonization is evident (c”) in condyle of patient 4. Asterisks (*∗*) indicate the articular surfaces of condyles. Scale bar = 20 *μ*m.

Figure 2 shows the preoperative CT images (a–c) and the results of hematoxylin and eosin (a'–c') and Gram (a”–c”) staining of bone specimens from patient 3. Severe bone destruction, inflammation, and bacterial colonization were evident in all areas of the specimens (mandibular body, Figure 2(a') and (a”); ramus, Figure 2(b') and (b”); and condyle, Figure 2(c') and (c”)).  Figure 3 shows the preoperative CT images of condyle (a–c) and the results of hematoxylin and eosin (a'–c') and Gram (a”–c”) staining of mandibular condyle specimens from patients 1, 2, and 4. Bacterial colonization was detected in the mandibular condyles of patients 1 (Figure 3(a”)) and 2 (Figure 3(b”)) but not patient 4 (Figure 3(c”)).

## 4. Discussion

Advanced mandibular ORN requires radical resection, such as hemimandibulectomy. In the current study, preoperative CT showed that three patients with severe mandibular ORN had cortical osteolysis extending to a location near the mandibular condyle (patients 1, 3, and 4). In patient 4, bacterial colonization was not evident in the mandibular condyle. In contrast, the lateral side of the ramus cortex did not exhibit osteolysis in preoperative three-dimensional CT in patient 2 (Figure 1b), although this patient exhibited bacterial colonization in the mandibular condyle. These results imply that preoperative CT findings may not reliably predict bacterial colonization in mandibular bone marrow. Moreover, the results show the potential usefulness of evaluating bacterial colonization by postoperative Gram staining to confirm the extent of resection.

### 4.1. Bacterial Colonization in the Mandibular Condyle

Some studies have investigated infectious disease in the mandibular condyle. Infections of the mandibular condyle can have hematogenous origin, spread from an adjacent structure, or be caused by direct inoculation [9]. A literature review reported that the etiologies of mandibular condylar infection in 20 patients were mandibular or maxillary molar extraction, pericoronitis, or ectopic third molar in eight patients; tuberculosis in four patients; malignant otitis externa or ear infection in four patients; and unknown in four patients [9]. In another study concerning bacterial colonization in the mandibular condyle of 25 patients with advanced osteoarthritis of the temporomandibular joint, bacterial colonization was not detected using the Gram stain technique; however, microorganisms grew in five broth cultures inoculated with swabs from mandibular condylar specimens [10]. The current study successfully revealed bacterial colonization by means of Gram staining; this technique may also be useful for the assessment of resection extent. Evaluation of bacterial colonization and resection margins using the Gram stain technique may aid in the assessment of resection extent and guide treatment planning after surgery; notably, long-term antibiotic administration may be recommended in patients with residual infection after surgery [11].

### 4.2. Possible Bacterial Spread to the Mandibular Condyle

An understanding of the mechanisms underlying bacterial spread is important for infectious disease control. The current study evaluated the areas of macroscopic bone destruction in coronal serial sections to determine how bone destruction might develop due to radiation damage and infection. In all patients, the areas of intraoral exposed bone were molar regions. Notably, the most apparent blackened degeneration was found in the medial sides of the cortical wall (Figure 1(a')–(d')). This result implies that the most vulnerable areas in patients with mandibular ORN are near the medial sides of the cortical wall within the mandibular body. In the mandible, the buccal cortex in premolar, molar, and retromolar regions has been described as the most vulnerable site for radiation-induced vascular disease and periosteal damage [12]. The posterior mandible has a compact and dense nature that is presumably related to the reduced vascularity of these regions; the higher mineral content of the bone may also cause greater absorption of radiation [13]. Furthermore, a combination of periosteal and endosteal blood supplies is present in the mandible. The cortical blood supply in molar regions was mainly periosteal and relatively poor, compared with the main endosteal supply to the mandibular condyle [14]. The current findings suggested that the invasion of bacteria originated from areas of intraoral exposed bone and extended posteriorly through the most vulnerable areas (i.e., medial side of the cortical wall) towards the mandibular condyle. This finding implies that the bacteria move toward areas of greater blood flow. As shown in Figure 1 (especially Figure 1b), destruction and bacterial colonization may occur in the medial side of the wall, although three-dimensional CT images do not clearly demonstrate apparent cortical destruction. Clinicians should carefully consider this possible underlying pathology of severe mandibular ORN.

Although all of our patients suffered from cutaneous skin fistulae, cutaneous skin fistula is not a cause but a result of intraoral infection. Therefore, bacteria colonized within the mandibular condyle probably originated from oral cavity. Origin of bacteria within the mandibular condyle can be identified with polymerase chain reaction, so further researches are necessary.

It is noted that the dissemination process of bacteria is probably not time-dependent. The occurrence timing of ORN as well as pathological fracture and cutaneous skin fistula formation is so diverse in each patient. The dissemination of bacteria probably associates with pathological fracture and poor oral hygiene. Early occurrence of pathological fracture resulting in short time interval between RT and surgery was found in patient 2 who received intensity-modulated radiotherapy. Patient 4 suffered from severe bone destruction, but no bacterial colonization within the mandibular condyle was found. Strikingly, he was relatively younger than other patients and had better oral hygiene and dental status as shown in Figure 1(d). The association between occurrence timing of pathological fracture, dissemination process of bacteria, oral hygiene, and RT type should be investigated in future too.

### 4.3. Potential Prevention and Management of Bacterial Colonization within the Condyle

In the management of ORN, the elimination of oral or dental infection before initiation of RT is a critical consideration [15]. Although the conservative management of mandibular ORN is also an important option [16], given current expertise in microvascular surgery, the use of well-vascularized free tissue transfer is needed for patients with advanced mandibular ORN who require extensive debridement leading to large composite defects. This approach can allow functional restoration with optimal cosmesis, while providing nonradiated soft tissue coverage with an intact blood supply [13]. The findings in this study suggest that antibiotics may not reach areas with poor vascularity (e.g., medial side of the wall of the mandibular body and ramus, or within the mandibular condyle). A well-vascularized flap improves blood supply to the defect region, thus promoting healing and potentially enhancing the viability of the remaining bone, despite the presence of residual ORN [17].

In clinical practice, removal of the mandibular condyle during extensive debridement of advanced mandibular ORN remains a focus of extensive debate. However, if bacteria are present within the mandibular condyle, regardless of osteosynthesis implementation, transferred tissue with robust vascularization may lead to enhanced bacterial activity. Although this study included a small number of patients, the findings indicate that the presence of bacteria within the mandibular condyle should be carefully considered in treatment of patients with advanced mandibular ORN. One of the limitations of this study is that we performed only macroscopic analysis, hematoxylin and eosin, and Gram staining, whereas polymerase chain reaction is required for bacterial identification. Another limitation was that bacterial spread outside of the mandible (e.g., subperiosteal spread) could not be assessed in this study. Further investigations with larger samples are needed to analyze bacterial spread both inside and outside of the mandible.

## 5. Conclusions

Our findings demonstrated bacterial colonization in the condyle of patients with advanced mandibular ORN. Infection was presumed to spread posteriorly through the most vulnerable areas in mandible (i.e., the medial side of the wall) in patients with ORN, which could be an important consideration in clinical treatment and prevention of ORN.

## Figures and Tables

**Table 1:** Clinical characteristics of patients with advanced mandibular osteoradionecrosis.

Patient	Sex	Age (years)	Pathological diagnosis (primary site)	RT^c^ type and dose	Interval between RT and surgery	ND^e^	CT^f^	Location of bone exposure	Pathological fracture	Cutaneous skin fistula	Preoperative IAN^g^ paralysis
1	Male	76	SCC^a^ (right nasopharynx)	Conventional 70 Gy	201 months	No	Yes	Left molar	Yes	Yes	Yes

2	Male	70	SCC^a^ (right oropharynx)	IMRT^d^ 69.96Gy	43 months	No	Yes	Right retromolar	Yes	Yes	Yes

3	Male	82	SCC^a^ (right oropharynx)	Conventional 60 Gy	157 months	No	No	Right molar-retromolar	No	Yes	Yes

4	Male	57	UC^b^ (left nasopharynx)	Conventional 70 Gy	155 months	No	Yes	Right retromolar	No	Yes	Yes

Abbreviations: ^a^SCC, squamous cell carcinoma; ^b^UC, undifferentiated carcinoma; ^c^RT, radiotherapy; ^d^IMRT, intensity-modulated radiotherapy; ^e^ND, neck dissection; ^f^CT, chemotherapy; ^g^IAN, inferior alveolar nerve.

**Table 2:** Extents of macroscopic bone destruction and bacterial colonization within the condyle in hemimandibulectomy specimens.

Patient	Macroscopic bone destruction							Bacterial colonization
	Mandibular body (anterior to bone exposure *∗*)		Bone exposure area		Ramus (posterior to bone exposure)		Condyle	Condyle
1	−	−	Crest side	+	Around MC^a^	++	+	+
					Near inferior margin	++		

2	−	−	Crest side	+	Around MC^a^	+	−	+
					Near inferior margin	++		

3	Near extraction socket	+	Crest side	++	Around MC^a^	++	++	++
					Near inferior margin	+		

4	−	−	Near inferior margin	++	Around MC^a^	++	−	−
					Near inferior margin	+		

Abbreviations: ^a^MC, mandibular canal. *∗* The most anterior bone sections are resected margins, so those are excluded for evaluation. The extents of macroscopic bone destruction and bacterial colonization are each classified into four grades: −, none; ±, little; +, moderate; ++, severe.

## Data Availability

The data used to support the findings of this study are available from the corresponding author upon request.
